# Liver resection for hepatocellular carcinoma in oldest old patients

**DOI:** 10.1186/s12957-018-1541-0

**Published:** 2019-01-03

**Authors:** Feng-Hsu Wu, Ching-Hui Shen, Shao-Ciao Luo, Jen-I Hwang, Wen-Shan Chao, Hong-Zen Yeh, Yee-Gee Jan, Yun Yen, Shao-Bin Cheng, Cheng-Chung Wu, Yi-Ling Lin, Fang-Ku P’eng

**Affiliations:** 1 Department of Surgery, Taichung Veterans General Hospital, Section 4, No. 1650, Taiwan Boulevard, Taichung, Taiwan; 2 Anesthesiology, Taichung Veterans General Hospital, Taichung, Taiwan; 3 Radiology, Taichung Veterans General Hospital, Taichung, Taiwan; 4 Gastroenterology, Taichung Veterans General Hospital, Taichung, Taiwan; 5 Pathology, Taichung Veterans General Hospital, Taichung, Taiwan; 6 Department of Surgery, School of Medicine, National Yang-Ming University, Taipei, Taiwan; 7 Cancer Translation Research Center, Taipei Medical University, Taipei, Taiwan; 8 Department of Surgery, Chung-Shan Medical University, Taichung, Taiwan; 9 Department of Surgery, Cheng-Hsin General Hospital, Taipei, Taiwan

**Keywords:** Hepatocellular carcinoma, Oldest old, Liver resection

## Abstract

**Background:**

For hepatocellular carcinoma (HCC), liver resection is a classical curative modality, despite its technical complexity. The incidence of HCC in the oldest old people (aged ≥ 85 years) is rising along with the global increase in life expectancy. Currently, no report has addressed liver resection for HCC in this aged population.

**Patients and methods:**

We conducted a retrospective review of 1889 patients receiving curative liver resection for newly diagnosed HCC from 1992 to 2016. At the time of operation, 1858 of them were aged < 85 years (group A), and 31 were aged ≥ 85 years (group B). Another 18 oldest old patients, whose HCC was considered resectable but were not operated on due to the patient’s refusal, served as the control group (group C). The clinicopathological characteristics and early and long-term outcomes were compared between groups A and B. All associated co-morbidities of the patients were well-treated before liver resection. The overall survival (OS) rates were also compared between groups B and C.

**Result:**

Group B had a significantly higher incidence of associated co-morbidities and hepatitis C infection. Postoperative complication rates and 90-day mortality rates after liver resection did not differ between groups A and B (*p* = 0.834 and *p* = 1.000, respectively), though group B had a longer postoperative stay (*p* = 0.001). In groups A and B, the 5-year disease-free survival rates were 29.7% and 22.6% (*p* = 0.163), respectively, and their overall survival rates were 43.5% and 35.5% (*p* = 0.086). The overall survival rate of group B was significantly different from group C (35.5% vs. 0%, *p* = 0.001).

**Conclusion:**

Despite a longer postoperative recovery period, liver resection for HCC in the oldest old patients may be justified if co-morbidities are well controlled.

## Background

Hepatocellular carcinoma (HCC) is a high lethal malignancy with increasing prevalence. As an endemic area of hepatitis B virus, hepatocellular carcinoma (HCC) has ranked as the first or second commonest cause of cancer death each year over the last 20 years [1]. During the past three decades, many modalities have been developed for treating HCC [1, 2]. Among them, liver resection, liver transplantation, and radiofrequency ablation (RFA) are generally agreed curative modalities for HCC [1–3]. Many non-operative modalities, such as trans-arterial chemo-embolization (TACE), trans-arterial radio-embolization (TARE), or target therapies have also been developed for HCC recently [2–4]. Although liver resection is the most classical modality, with a mortality rate of < 3%, it remains a complex procedure with high postoperative complication rates (20–40%). Liver resection would be better performed at high-volume centers with well-experienced surgical teams [1, 5]. Selection for the appropriate modality at the right time for the appropriate patient groups may improve the survival of HCC.

On the other hand, human life expectancy is increasingly lengthened due to improved medication and health care. The definition of “elderly people” four decades ago was those aged “65 years old” [6]. The new definition of elderly has been extended to people > 80 years old [7]. Older patients usually have a higher incidence of the associated co-morbidities, which are often serious [8–10]. Elderly people can be roughly subdivided into three groups, viz., “young old,” over 65 but under 75 years; “intermediate old,” over 75 but under 85 years; and “the oldest old,” over 85 years [7].

In 1990, Fortner et al. first demonstrated that despite higher risks of liver resection in elderly patients (age ≥ 65 years), liver resection remains possible after careful patient selection [11]. Thereafter, a number of other investigators reported the feasibility of liver resections for HCC in old patients [12–14]. Their definition of old age was from 65 to 70 [12] and even 80 [13, 14]. Liver resection for HCC in the oldest old patients has not been addressed so far. The main age of HCC incidence is 50 to 70 years. On the other hand, non-operative modalities, as described above, also develop. Although these treatments do not cure the HCC, they do prolong the survival times with acceptable life quality [3]. Thus, the actual benefits of liver resection for HCC in oldest old patients remain to be determined.

The life expectancy of the Taiwanese population has increased rapidly during the last 20 years. As a tertiary referral center, we sometimes encounter oldest old HCC patients. This age is over the average life expectancy worldwide and in Taiwan [15], so we have been hesitant about aggressive surgical intervention for this particular population. Some oldest old HCC patients are in relatively good general condition to undergo the operation, but their tolerance for surgical complications and benefit from operation, such as median survival time, are still unclear. To elucidate the benefits of liver resection for HCC in these particular patients, we conducted a retrospective review of prospectively collected data of liver resection for HCC over the past 25 years.

## Patients and methods

### Patient data

We undertook a retrospective review and analysis of the clinicopathological data of 1910 consecutive patients who had undergone liver resections for newly diagnosed HCC during the period from January 1992 to December 2016. Patients whose HCC resection needed cardiopulmonary bypass for tumor thrombus extending to the right atrium (*n* = 11) [16] and patients who received non-curative liver resection (*n* = 10, defined as gross residual tumor after operation [8]) were excluded.

Among all enrolled patients, 1858 of them were aged < 85 years at the time of operation (group A, median 62, range 18–84). The age of the remaining 31 patients was ≥ 85 years (group B, median 87.5 years, range 85–95). We then compared groups A and B regarding patients’ background features, tumor characteristics, and the early and long-term postoperative outcomes.

During the same study period, 18 oldest old patients were judged as resectable for HCC but were treated by non-operative modalities instead due to the refusal of operation by the patients or their families (group C). Their treatment modalities were RFA in 4, TACE in 9, oral sorafenib in 3, and conservative treatment in 2. TACE or RFA was performed by a senior radiologist (JIH).

Since July 2012, laparoscopic hepatectomy was performed on one patient in group B and 51 in group A. Tumors of these patients met the patient selection criteria of the Louisville statements of laparoscopic hepatectomy [17].

### Preoperative assessments

As we have previously reported [8], HCC patients underwent measurement of conventional hemogram and liver function tests: serum α-fetoprotein (AFP), hepatitis B surface antigen (HBsAg), and anti-hepatitis C antibody (anti-HCV); indo-cyanine green (ICG) clearance test; gastroduodenal endoscopy; abdominal computed tomography (CT); and/or magnetic resonance imaging (MRI). The resectability and the extent of the liver to be resected were based on the tumor extension and modified Makuuchi criteria [18].

Patients whose co-morbidities should be well treated and controlled and who fell into American Society of Anesthesiology (ASA) class one or two were considered operable. After 2001, forced expiratory volume at 1 s (FEV 1) ≥ 75% and left ventricular ejection fraction (LVEF) in echocardiogram ≥ 50% were added to the patient selection criteria in patients older than 65 years. The treatment strategies, management plans, and tentative operative procedures of each patient were agreed upon before operation in conferences attended jointly by surgeons, gastroenterologists, anesthesiologists, radiologists, and co-morbidity-related physicians. The associated co-morbidities were the following: cardiopulmonary diseases, including hypertension, heart failure, cardiomyopathies, valvular heart disease, pericardial disease, syncope, aortic aneurysms, coronary arterial disease, and arrhythmia; lung malignancies, including chronic obstructive pulmonary disease, bronchiectasis, obstructive sleep apnea, interstitial lung disease, and pulmonary hypertension; neuromuscular disorders, including seizure, myopathies, stroke, dementia, and Parkinson’s disease; gastrointestinal diseases, including peptic ulcer disease, malignancies other than HCC, inflammatory bowel disease, gastrointestinal bleeding, biliary tract diseases, esophageal and gastric disorders; endocrine and metabolic diseases, including breast disease, thyroid disease, adrenal disorders, pituitary disorders, diabetes mellitus, and dyslipidemia; urologic and nephrologic diseases, including end-stage renal disease, urologic and genital malignancies, lithiasis, renal failure and glomerular disease; hematologic diseases, including anemia, platelet disorders, coagulopathies, leukemias, lymphomas and myeloproliferative disease; and rheumatologic diseases, including systemic lupus erythematosus, vasculitis, amyloidosis, various arthritis, and other autoimmune diseases. For patients with end-stage renal disease, peri-operative heparin-free hemodialysis was carried out [19]. For patients with hypersplenic thrombocytopenia (platelet count ≤ 80,000/mm^3^), concomitant splenectomy was carried out [20]. For patients with severe gastroesophageal varices (F3 or presence of red-colored sign), we performed pre-operative endoscopic ligation and/or sclerotherapy [21]. In addition to post-operative pain control, epidural catheter insertion for analgesic injection was performed routinely after 2005.

### Intraoperative assessments

Liver parenchyma was transected using a Kelly crushing method under intermittent hepatic inflow blood occlusion (Pringle’s maneuver). During liver parenchymal transection, a low central venous pressure (CVP) policy (CVP < 5 cm H_2_O) was implemented by a senior anesthesiologist (CHS), who also oversaw the restrictive blood transfusion policy.

### Postoperation assessment

Resected specimens were examined by a senior pathologist (YGJ), who determined the following: tumor capsular formation, resection margin width, tumor number, micro- or macro-vascular invasion, Ishak score, cirrhosis severity [22], and tumor differentiation (using Edmondson and Steiner grading). The AJCC Cancer staging system (8th edition) was applied after pathological examinations. Early postoperative complications, such as bile leakage, ascites, and liver failure, were recorded as defined according to the international consensus [23–25]. The severity of complications [26] was determined using the Clavien-Dindo classification. Any death that had occurred within 90 days after the operation was considered operative mortality.

Patients who survived from hepatectomy were followed up at the outpatient clinic during the first 2 years at intervals of 2 to 3 months, and thereafter at intervals of 4–6 months. Serum AFP, liver function, and imaging (abdominal ultrasonography, CT, or MRI) were checked. Recurrent HCCs were treated by re-hepatectomy, RFA, TACE, oral sorafenib, or other conservative treatments, as appropriate.

### Statistical analysis

Continuous variables are expressed as median (range) and were compared using Mann-Whitney *U* test. Frequencies were compared using Fisher’s exact test or Pearson’s *χ*^2^ test. All patients were followed up till July 2018. The disease-free survival rates and overall survival rates were characterized by the Kaplan-Meier life-table method and compared using the log-rank test. *p* values < 0.05 were considered statistically significant.

## Results

### Patient background characteristics

Table 1 shows the clinicopathological characteristics of patients with resected HCC (groups A and B) and clinical features of the oldest old HCC patients without surgery (group C). Groups B and C had significantly higher incidences of associated co-morbidities and anti-HCV positivity compared with group A. Clinicopathological characteristics did not significantly differ between groups B and C.

**Table 1:** Clinicopathological features in the three groups

Clinical characteristics	Group A (*n* = 1858)	Group B (*n* = 31)	Group C (*n* = 18)	*p* value	
				A vs. B	B vs. C
Sex (M:F)	1421:437	25: 6	11:7	0.741	0.102
Age (years)	63 (18–84)	86 (85–95)	87 (85–92)	< 0.001	0.914
Serum hepatitis states				0.024	0.894
B + C+	108 (5.8%)	1 (3.2%)	0		
B + C−	873 (47.0%)	11 (35.5%)	5 (27.8%)		
B − C+	590 (31.8%)	13 (41.9%)	10 (55.6%)		
B − C−	287 (15.4%)	6 (19.4%)	3 (16.7%)		
Serum AFP (ng/ml)	30.9 (0.63–3,395,610)	28.3 (1.29–15,321)	34.6 (12.0–127,911)	0.814	0.758
ICG 15 (%)	13.5 (1.38–59.65)	15.3 (6.60–29.53)	14.2 (5.4–38.0)	0.814	0.758
Child-Pugh grade					
A:B:C	1612:205:41	27:3:1	16:2:0		
Need for splenectomy	141 (7.5%)	1 (3.2%)	1 (5.3%)	0.190	0.711
Associated with EGV	800 (16.0%)	4 (12.9%)	2 (11.1%)	0.584	0.899
Associated with comorbidities	454 (24.3%)	29 (93.8%)	16 (88.9%)	< 0.001	0.916
Cardiopulmonary	201	20	12		
Neurologic	10	2	0		
Hepatico-gastroenterologic	66	4	0		
Endocrine and metabolic	125	6	6		
Genitoenphrologic	69	3	1		
Hematologic	72	0	0		
Rheumatologic	19	3	0		
Others	41	2	2		

Hepatitis states: B + C+, positive for HBsAg and anti-HCV; B + C−, positive for HBsAg, negative for anti-HCV; B− C+, negative for HBsAg and positive for anti-HCV; B− C−, negative for HBsAg and anti-HCV; *AFP* α-fetoprotein, *ICG 15* indocyanine-green 15-min retention test, *EGV* esophagogastric varice

### Operation result and early outcome

Group B had a significantly longer postoperative hospital stay compared with group A [18.0 (8–46) days vs. 10.0 (7–81) days, *p* = 0.001]. No significant differences were found in intra- or early post-operative results between the two operated groups (Table 2).  The pathologic features between groups B and A have no significant difference (Table 3).

**Table 2:** Intra- and early-postoperative result in hepatectomy for HCC

	Group A (*n* = 1858)	Group B (*n* = 31)	*p*
Operative time (hour)	4.6 (3.2–14.9)	4.0 (0.8–10)	0.282
Liver ischemic time (min)	30.0 (14.8–204)	28.8 (17.1–94.3)	0.282
Liver transection area (cm2)	36.8 (2.4–164.0)	42.6 (16.0–120.0)	0.564
Operative bleeding (ml)	520 (20–10,024)	600 (50–1900)	0.395
Need for blood transfusion	329 (17.7)	6 (19.4%)	0.994
Postoperative stay (day)	18.0 (5–46)	10 (7–81)	0.001
Postoperative complications	361 (19.4%)	7 (22.6%)	0.834
90-day mortality	17 (0.91%)	0	1.000
Clavian-Dindo grade			
Grade III	196	3	
Grade IV	62	1	
Grade V	16	0	
Post-operation pneumonia	2	0	
Pleural effusion	31	0	
Ascites	38	0	
Bile leakage	101	1	
Liver failure	16	0	
Delay bowel movement	13	3	
Wound infection	27	1

**Table 3:** Pathologic characteristics of resected specimen

Pathological features	Group A (*n* = 1858)	Group B (*n* = 31)	*p*
Tumor size (cm)	6.5 ± 5.1	5.6 ± 5.7	
Histological characteristics	1414 (75.7%)	22 (74.0%)	0.691
Tumor number ≥ 2	352 (18.9%)	4 (12.9%)	0.540
Microvascular			
Invasion	787 (42.1%)	12 (38.7%)	0.848
Satellite nodule	608 (32.5%)	9 (29.0%)	0.831
Capsule formation	961 (51.3%)	21 (67.7%)	0.103
Resection margin (mm)	3.0 (0–19.0)	1.0 (0–65)	0.961
Tumor differentiation			
Well differentiation	308 (16.6%)	3 (9.7%)	0.546
Moderately differentiation	450 (24.2%)	9 (29.0%)	
Poorly differentiation	1100 (59.2%)	19 (61.3%)	
AJCC-TNM stage			0.936
I	739 (39.8%)	12 (40.0%)	
II	556 (29.9%)	10 (33.3%)	
III	548 (29.5%)	9 (29.4%)	
IV	15 (0.8%)	0 (0%)	

### Long-term result

**Fig. 1:**
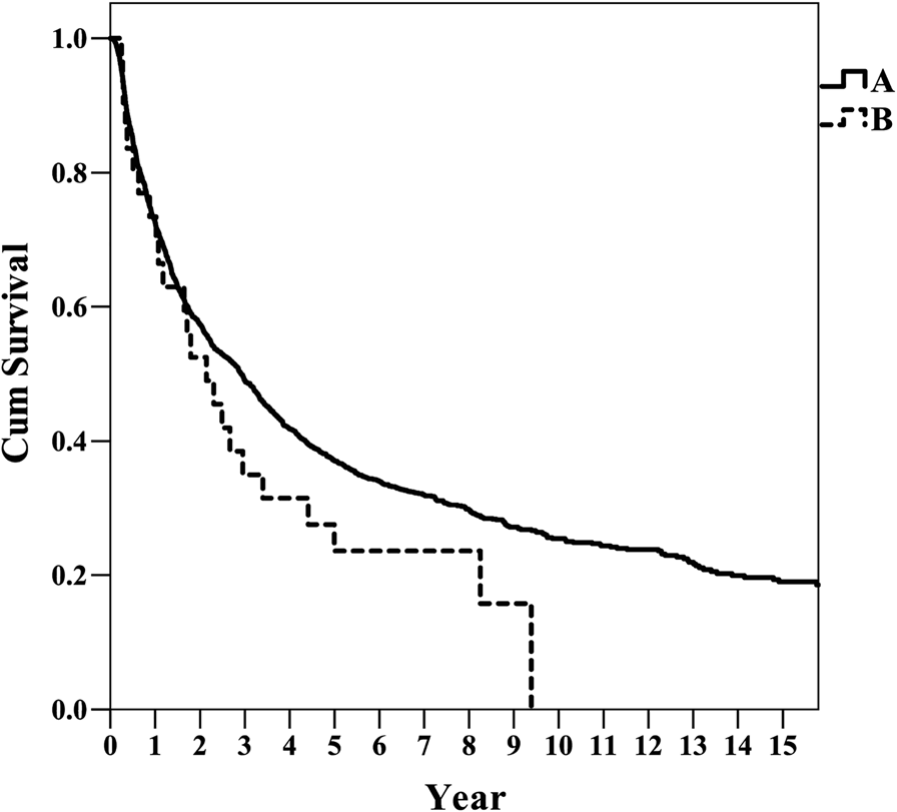
Disease-free survival rates (DFS) in groups A and B, *p* =0.163

**Fig. 2:**
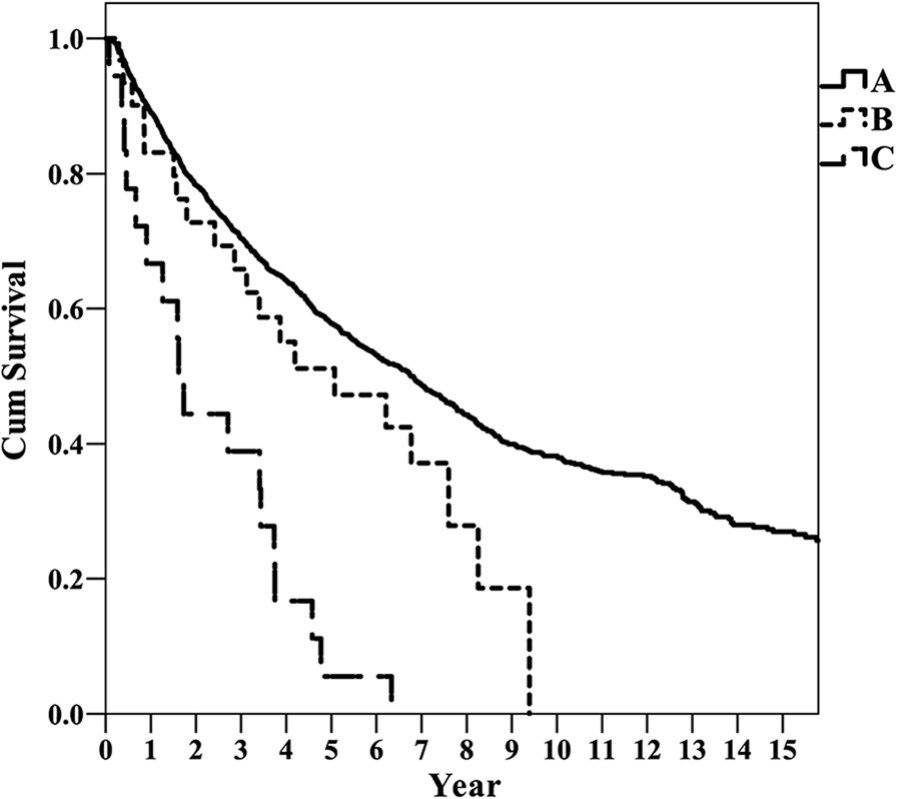
Overall survival curves in groups A, B, and C. *p* value for A vs. B, 0.080; B vs. C, < 0.001

The difference in DFS between groups B and A was not significant (*p* = 0.163) (Fig. 1). The OS of Group B was slightly but not significantly lower than that of group A (*p* = 0.086). On the other hand, group C had very poor outcomes compared to group B (Fig. 2).

A total of 1098 patients in group A died during the course of this study. Among them, deaths of 105 patients were not due to HCC, and the remaining 993 patient deaths were HCC-related. In contrast, only 20 patients in group B died, 6 of them due to other diseases (*p* < 0.001). The median survival times of group A, B, and C were 6.9, 5.4, and 1.9 years, respectively.

## Discussion

In Taiwan, the average life expectancy had exceeded 80.0 years by 2014 (mean 80.0 years; male 76.8 years; female 83.4 years in 2016). People aged ≥ 65 years constitute 13.3% of the population, and the country’s aging index (the number of people ≥ 60 years old per 100 people < 15 years old) was 100.18 in 2016 [15]. This means increasing population aging is impending, mirroring the global trend. The remaining life expectancy of 65-, 75-, and 85-year-olds was 19.9, 12.6, and 6.8 years, respectively [15].

The elderly population has a higher risk of HCC formation [27]. In our daily clinical practice, increasingly more elderly subjects have been diagnosed with HCC. Our previous study explored the potential benefits of hepatectomy in octogenarians [8]. Since then, a number of studies have reported on the feasibility of liver resection to treat HCC in old patients. Uwatoko et al. [28] reported successful resection of HCC in two patients aged > 90 years, but they did not present long-term outcomes. To our best knowledge, ours is the first study regarding the outcomes of hepatectomy in oldest old patients.

This study is a single-institution observation of HCC resection with a relatively large sample observed over a relatively long period of time. The liver resection strategy and surgical procedure were homogenous across patients. Surgical indications, operation methods, and follow-up policies were all similar during the study period. Furthermore, an anesthesiologist, radiologist, gastroenterologist, and co-morbidities-related physician jointly discussed the treatment options preoperatively. Our average case intake was approximately 75 cases/year in volume, which is considered high for HCC resection in hospitals [5, 29].

During the study period, we have improved on the surgical equipment and techniques, concepts, and medication prescriptions [30] to treat early surgical complications. The positive trend of long-term outcomes of our liver resections for HCC has noticeably risen with time.

Our group B and C patients had higher incidences of co-morbidity and hepatitis C infection. These results are consistent with previous studies [27, 31]. Furthermore, poor organ function and liver function in the elderly have been reported in the early literature [32]. Yamada et al. [33] showed that old patients (> 80 years) had lower serum albumin than younger patients. Okinaga et al. [29], on the other hand, reported that the number of patients with multiple co-morbidities dropped in patients aged > 80 years compared with younger patients (70–80 years).

In a previous study, we used the ASA score as the sole criterion for patient selection. One 84-year-old patient expired due to acute myocardial infarction 3 weeks after the HCC resection. Therefore, in the present study, we added more criteria for patient selection: FEV1 ≥ 75% and LVEF ≥ 50% for those aged ≥ 65 years. No postoperative deaths had occurred under the screening after 2004 with these new criteria.

Age is a risk factor of post-liver resection pulmonary complication. Kim et al. [34] reported that older patients (> 70 years of age) had higher incidences of postoperative pneumonia. Here, we found no severe pulmonary complication in the older group. Our pre-operative cardiopulmonary function test, used as a patient selection criterion, and respiratory training with incentive spirometer and postoperative pain control were arranged to exclude the possibility of pulmonary complications. In addition, we noted that older patients required longer periods for postoperative recovery without increasing the complication rate. We believe that this finding was related to the longer recovery of postoperative ileus in the older group [8].

Nozawa et al. [9] also reported a higher incidence of postoperation cardiovascular complication and delirium in super-elderly patients (aged > 80 years). Apart from this, no difference in liver-associated postoperative complications was found between the older and younger patients [29]. We used a pre-operative echocardiogram and LVEF as additional criteria for patient selection. Postoperative ileus, delirium, cardiovascular disease, and pulmonary complications should contraindicate liver resection surgery in patients aged ≥ 70 years [35]. Our multi-departmental consensus conference on the treatment modalities for each HCC patient helped to support the feasibility and safety of hepatectomy for elderly patients, including those ≥ 85 years.

Regarding the long-term survival after HCC resection, most studies reported no difference in OS or DFS between older and younger patients [9, 32, 34, 36–38] (Fig. 2). Here, we also found no significant differences between the older and younger group, though in the older group, we found a tendency of shorter OS. Furthermore, we also observed that the operated oldest old patients had better prognosis than the non-operated control group. According to these results, we believe that hepatectomy for HCC has long-term benefits, consistent with previous studies.

For late deaths in our oldest old operated patients, we observed fewer HCC-related deaths compared with the younger patients. Nozawa et al. [9] also reported that older patients who underwent hepatectomy for HCC had fewer cancer-related deaths than younger patients. On the other hand, we found that all of non-operated oldest old patients succumbed to HCC. Toro et al. [3] assessed life quality within 2 years after hepatectomy for HCC, finding that it was better than those who underwent RFA, TACE, or no treatment. Therefore, regarding HCC treatment and quality of life, liver resection is mandatory in well-selected oldest old patients.

The current study showed that median survival time after liver resection for HCC in group B (5.4 years) was approximately 1.4 years lower than the life expectancy of the general oldest old population (6.8 years) in Taiwan. In contrast, the median survival time of group C was 1.9 years (4.9 years below the life expectancy). We concluded that liver resection for HCC in oldest old patients might let these patients be able to achieve their natural life expectancy without viable HCC, a lethal malignancy.

There are some limitations to this study. First, this is a cohort study, and many perioperative assessments, postoperative care schedule, and strategies of treatments were not fully consistent during the whole period of study. Second, our strategy could have changed with some biases and with time. Finally, we provided no randomized comparisons of other medical non-operative modalities (e.g., RFA, TACE, or TARE) due to the small sample size.

Despite these limitations, we can still conclude that liver resection for HCC may be justified in highly selected oldest old patients with well-controlled health conditions.

## Conclusion

Despite a longer postoperative recovery period, liver resection for HCC in the oldest old patients may be justified if co-morbidities are well controlled.
